# Enriched environment alleviates stress-induced dry-eye through the BDNF axis

**DOI:** 10.1038/s41598-019-39467-w

**Published:** 2019-03-04

**Authors:** Kokoro Sano, Motoko Kawashima, Toshihiro Imada, Toru Suzuki, Shigeru Nakamura, Masaru Mimura, Kenji F. Tanaka, Kazuo Tsubota

**Affiliations:** 1 Department of Ophthalmology, Keio University School of Medicine, Tokyo, 160-8582 Japan; 2 Department of Neuropsychiatry, Keio University School of Medicine, Tokyo, 160-8582 Japan

## Abstract

The number of patients with dry eye disease (DED) is increasing, and DED has become an urgent public health problem. A comorbidity of mental disorders has been reported in DED patients. We hypothesized that physical and psychological stressors impair tear secretion. To examine the relationship between stress loading and decreased tear secretion, we established a stress-induced DED mouse model, which permitted us to address the underlying mechanism of pathogenesis and resilience. Enriched environment (EE) was an effective intervention to prevent and alleviate stress-induced decreased tear secretion. Because stress loading resulted in decreased brain-derived neurotrophic factor (BDNF) expression while EE resulted in increased expression, we focused on the role of BDNF in tear secretion. Using two distinct *Bdnf* gene knockdown mice, we evaluated whether BDNF was a deterministic factor in regulating tear secretion in healthy and stressed conditions. *Bdnf* knockdown mice showed decreased basal tear secretion and loss of stress tolerance by EE for tear secretion. These results suggest that BDNF expression is related to tear secretion and to the pathology of DED.

## Introduction

Dry eye disease (DED) is a complex multifactorial disease of the tear film and ocular surface that results in a lack of tear secretion. DED has become a serious problem in modern society. In particular, visual display terminals (VDTs), which are used by many office workers, have been shown to suppress blinking, which can increase evaporation and induce dry eye^1–5^. DED symptoms consist of various subjective perceptions, including pain, dryness, burning, and visual disturbances. Thus, DED negatively impacts patient quality of life^6,7^. It has previously been shown that systemic and physical conditions, such as diabetes, affect DED symptoms^8^. Moreover, several population-based studies have reported a relationship between psychiatric disorders and DED, including depressive disorder, anxiety disorder, sleep disorder, and post-traumatic stress disorder^9–16^; however, a causal relationship between the two has not been established.

We previously reported that continuous air blow to the eyes decreases basal tear secretion in rats^17^. Building on these results, we sought to develop a mouse model with chronic decreased tear secretion. In the current study, we developed a mouse model and examined its validity. We then addressed the direct relationship between physical/ psychological stressors and decreased tear secretion.

Establishing a mouse model with a DED-like phenotype permitted us to investigate therapeutic interventions as well as the molecular mechanisms underlying pathogenesis and resilience. Enriched environments (EEs) have been reported to alleviate disease symptoms and progression in Alzheimer’s disease, Parkinson’s disease, and cancer^18–20^. In the current study, we sought to determine whether an EE prevents or alleviates stress-induced DED-like symptoms in mice as well as the mechanisms by which any beneficial effects occur.

Changes in brain-derived neurotrophic factor (BDNF) activity in the brain have been proposed to be the predominant mechanism by which an EE provides beneficial effects on health^21,22^. Further, BDNF has been reported to be involved in stress-related symptoms^23–25^, and a *Bdnf* gene polymorphism has been associated with DED in humans^26^. Although the fundamental mechanism of BDNF’s role in tear secretion remains unclear, we hypothesized that a stress-related reduction in BDNF can cause DED-like symptoms in mice and that EE-related BDNF upregulation can alleviate these symptoms.

Transcription of the mouse *Bdnf* gene is controlled by at least 9 distinct promoters. Each promoter drives the expression of a small, untranslated exon spliced onto a common, final exon (exon IX), resulting in 9 different transcripts that encode the pre-pro-BDNF protein. Transcripts from exons I, II, IV, and VI (which encode isoforms 1, 2, 4, and 6, respectively) constitute the majority of *Bdnf* mRNA produced in the brain, while the transcript from exon VI is the major isoform outside of the brain^27,28^. Individual knock-out studies of these unique transcripts have highlighted the biological significance of alternative *Bdnf* transcripts^29,30^. Therefore, it is desirable to address the role of each respective *Bdnf* isoform on tear secretion. Here, we employed the FAST-system^30^ to generate isoform 1–3 knockdown mice and isoform 4–7 knockdown mice. Using these loss-of-function mouse lines, we investigated BDNF’s role in tear secretion.

## Results

### Stress causes decreased tear secretion in a mouse model of DED

**Figure 1:**
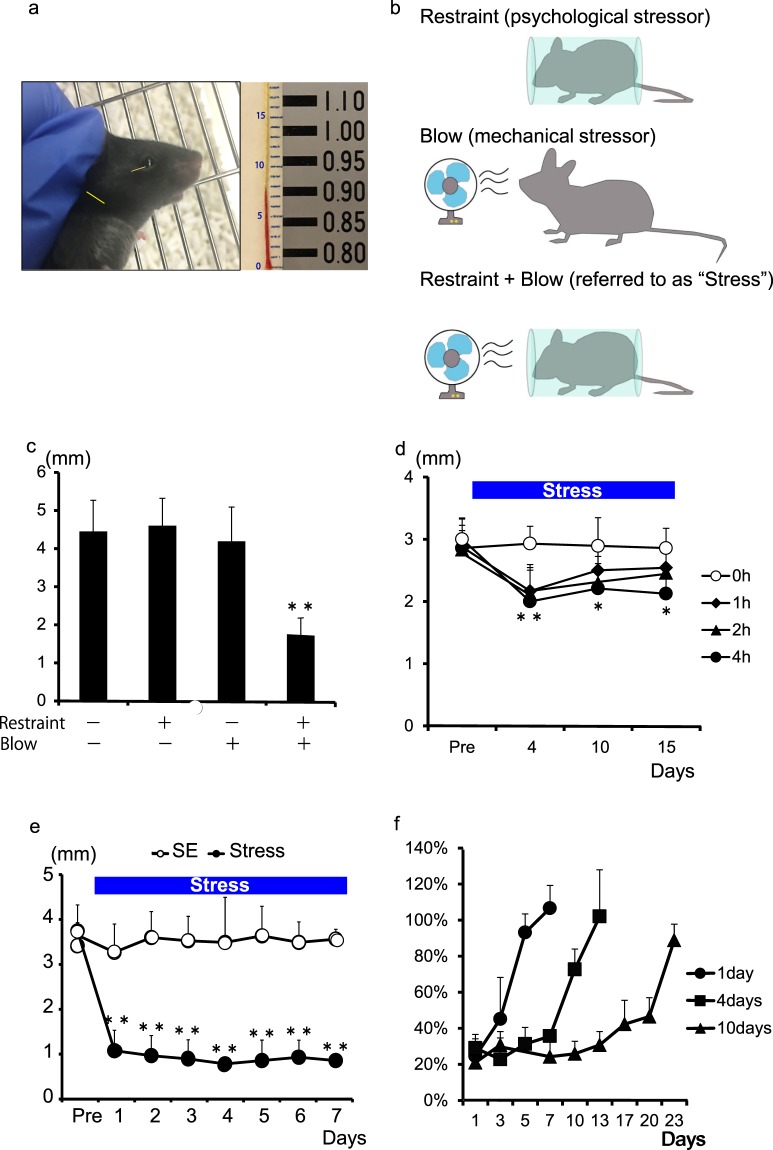
Stress decreases tear secretion. (**a**) Measurement of tear secretion volume using cotton thread test. The sample image shows 7. 9 mm length; 7 mm by a ruler (middle) and 0.9 mm by a scale (right). (**b**) Model of stress loading. (**c**) Tear secretion significantly decreased in response to restraint and blowing (n = 5; **P < 0.01 by one-way ANOVA with post-hoc Tukey’s test). (**d**) Within 15 days, the groups with 1 or 2 hours of stress showed recovery regardless of stress loading, while the group with 4 hours of stress maintained lowered tear secretion (n = 6–7; **P < 0.01, *P < 0.05 by one-way ANOVA with post-hoc Tukey’s test). (**e**) Four hours of stress was necessary to maintain reduced tear secretion (n = 6; **P < 0.01 by Student’s t-test). (**f**) Longer duration of stress loading resulted in a longer durations of maintenance and time required for recovery (n = 4–5).

To explore what types of stressors reduce the levels of basal tear secretion in mice, we examined basal tear secretion volume changes (Fig. 1a) after introducing psychological and/or mechanical stressors. We employed 4-hour behavioral restraint as a psychological stressor and 4-hour continuous blowing toward the eyes as a mechanical stressor^31^ (Fig. 1b). Neither restraint nor blowing changed basal tear secretion levels over the course of 7 days, but a combination of the two resulted in decreased tear secretion (Fig. 1c) and corneal damage as has been reported previously^31^. Hereafter, we refer to the combination of restraint and blowing as “stress”.

We then sought to establish the duration of loading stress required to reduce basal tear secretion and to maintain the lowered level. Mice were divided into 4 groups: 3 stress groups and 1 control group. The 3 stress groups were subjected to stress for 1, 2, or 4 hours per day, respectively. Initially, all stress groups showed a significant decrease in tear secretion compared to the control group over the course of 4 days (1 and 2 hours per day, p < 0.05; 4 hours per day, p < 0.01). However, the groups subjected to 1 or 2 hours of stress per day showed spontaneous recovery over the course of 15 days regardless of concurrent stress loading (Fig. 1d). In contrast, the group subjected to 4 hours of stress per day maintained lowered tear secretion over the same time period (p < 0.05). These data demonstrate that 1 hour of stress loading per day was sufficient to induce an acute reduction of tear secretion but was insufficient to maintain the reduction over time and that reduced tear secretion was maintained in response to 4 hours of stress per day (Fig. 1e).

After establishing that stress induces decreased tear secretion, we sought to determine how long decreased secretion lasts after stress withdrawal. To address this question, mice were loaded with stress for 4 hours per day for 1, 3, or 10 days, respectively, and tear secretion was measured after discontinuing the stress. We observed that decreased levels of tear secretion (less than 40%) lasted for 2, 6, and 18 days, respectively. Recovery to a 90% level occurred at 4, 10, and 22 days after discontinuing stress, respectively. These data indicate that longer stress loading results in a prolonged decrease of tear secretion and a longer time required for recovery (Fig. 1f).

The mice did not show a change in lacrimal gland (LG) weight regardless of decreased tear secretion (Supplementary Fig. 1a), and body weight did not change as a result of stress loading (Supplementary Fig. 1b). Altogether, these results demonstrate that physical and psychological stressors cause decreased tear secretion in the absence of LG atrophy and body-weight loss. Further, these results show the establishment of a chronic, reversible, stress-induced DED mouse model.

### Enriched environment prevents and alleviates decreased tear secretion in mice

**Figure 2:**
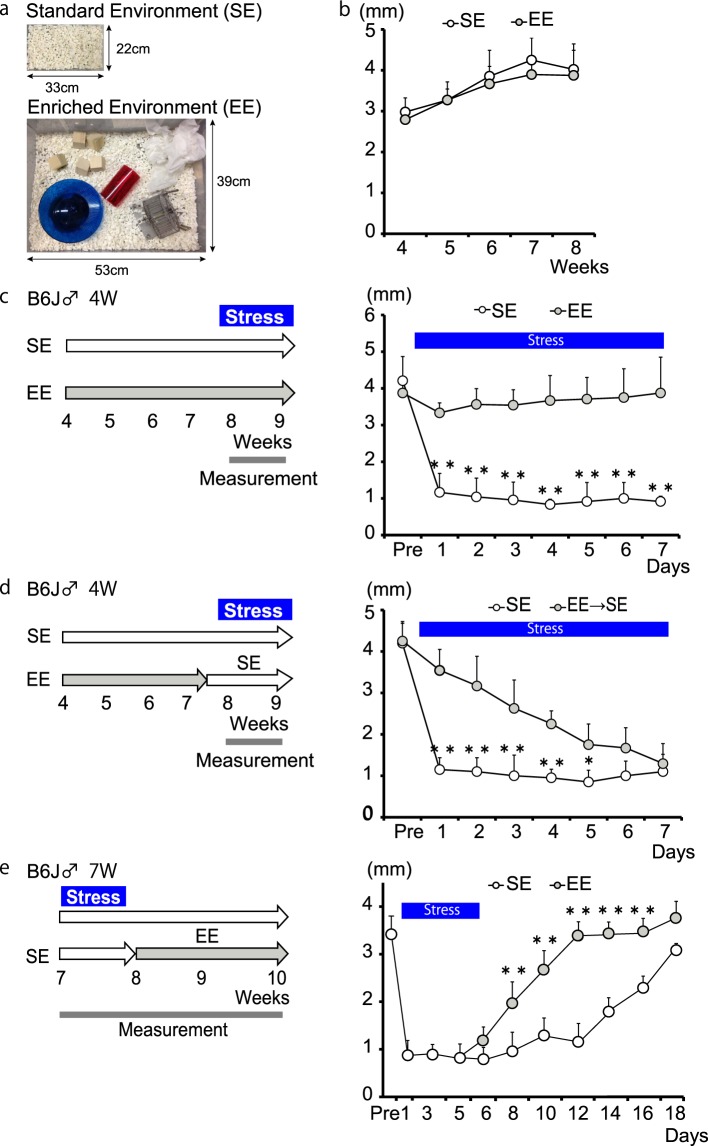
Enriched environment prevents and alleviates stress-induced decreased tear secretion. (**a**) Standard environment (SE) and enriched environment (EE) (**b**) Basal tear secretion was not affected by EE housing (n = 6). (**c**) EE housing prevented decreased tear secretion compared to SE housing (n = 6; **P < 0.01 by Student’s t-test). (**d**) EE prior to stress alleviated stress-induced decreased tear secretion (n = 6; **P < 0.01,*P < 0.05 by Student’s t-test). (**e**) EE introduced following stress was associated with faster recovery compared with SE housing (n = 6; **P < 0.01 by Student’s t-test).

We asked whether an EE could prevent or alleviate stress-induced decreased tear secretion. We used the standard definition of EE as a combination of complex inanimate objects (Fig. 2a).

Mice were divided into a standard environment (SE) housing group and an EE housing group. Housing began at 4 weeks of age and continued for 4 weeks. Basal tear secretion (Fig. 2b), LG weight (Supplementary Fig. 2a), and body weight (Supplementary Fig. 2b) were comparable in both groups, indicating that the EE did not affect general health condition or tear secretion. However, the EE completely offset the negative effects of stress on tear secretion (Fig. 2c). Furthermore, the EE prevented corneal damage by stress loading (Supplementary Fig. 2c).

We next examined how the timing of EE introduction affected the prevention or alleviation of stress-induced decreased tear secretion. When EE housing was introduced prior to stress, the abrupt, stress-induced decrease in tear secretion was prevented, and decreased tear secretion was mitigated (Fig. 2d, SE versus EE: after 1 day, p < 0.01; after 7 days, p < 0.01). When EE housing was introduced after the initiation of stress, the amount of time that it took the mice to reach 60% recovery was significantly shorter than that in the SE group (p < 0.01; Fig. 2e). Data from partially overlapping EE housing and stress supported the above findings. During stress loading, an EE to SE regimen was associated with decreased tear secretion (Supplementary Fig. 2d), and an SE to EE regimen resulted in a gradual recovery (Supplementary Fig. 2e). Taken together, these data indicate that an EE can abolish concurrent stress-induced decreased tear secretion and that an EE mitigates stress-induced decreased tear secretion when introduced before, after, and partially-overlapping with stress.

### BDNF expression levels decrease in response to stress and are restored in response to EE housing

**Figure 3:**
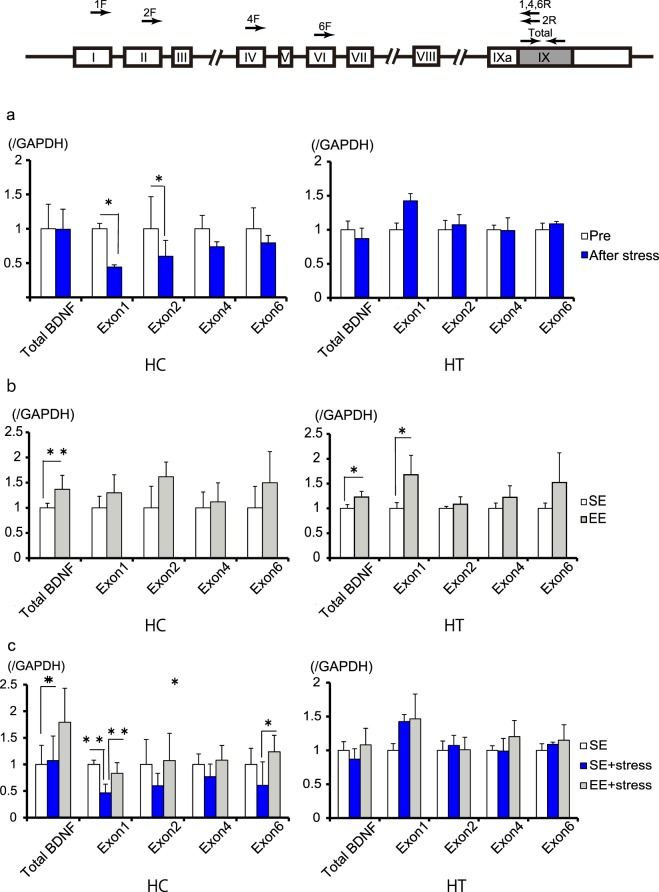
Bdnf expressions decreases in response to stress and increases in response to housing in an enriched environment. (**a**) In the hippocampus (HC), Bdnf expression decreased in response to stress (n = 3; *P < 0.05 by Student’s t-test). (**b**) In the HC and Hypothalamus (HT), Bdnf expression increased in response to an enriched environment (EE; n = 4; **P < 0.01, *P < 0.05 by Student’s t-test). (**c**) EE housing restored the previously-observed stress-dependent decline in Bdnf expression (n = 3; **P < 0.01, *P < 0.05 by one-way ANOVA with post-hoc Tukey’s test).

To elucidate the underlying mechanism of the relationship between tear secretion, stress, and EE, we measured the expression levels of the most abundant brain *Bdnf* transcripts (isoforms 1, 2, 4, 6, and total). We found that the levels of all isoforms in the hippocampus (HC) were decreased under stress conditions, though this change was only significant in exon I and II (exon I, p < 0.05; exon II, p < 0.05; total, exon IV, and VI, p > 0.05; Fig. 3a). We also observed that *Bdnf* transcripts were increased in the mice that had been exposed to an EE in the absence of stress (total, p < 0.01; not significant in others; Fig. 3b). Further, when stress was applied to mice exposed to an EE, a stress-induced decrease in *Bdnf* transcript levels did not occur, and transcript levels in the hypothalamus (HT) were increased (total p < 0.05; exon I p < 0.01; exon VI p < 0.05; not significant in exons II or IV; Fig. 3c).

*Bdnf* is also expressed outside of the brain. We used RT-PCR to assess *Bdnf* expression in the LG and detected only isoform 6. We quantitatively examined the levels of *Bdnf* mRNA in the LG under various conditions and found no significant differences in expression between the experimental groups (Supplementary Fig. 3). This suggests that *Bdnf* in the LG is unlikely to affect basal tear secretion under either stress or EE conditions. One caveat is that we did not assess the effect of BDNF derived from the peripheral nerves innervating the LG. However, we were able to demonstrate that the amount of brain BDNF was associated with the relationship between tear secretion, stress, and EE.

### Basal tear secretion is regulated by *Bdnf* isoforms 1 and 2

**Figure 4:**
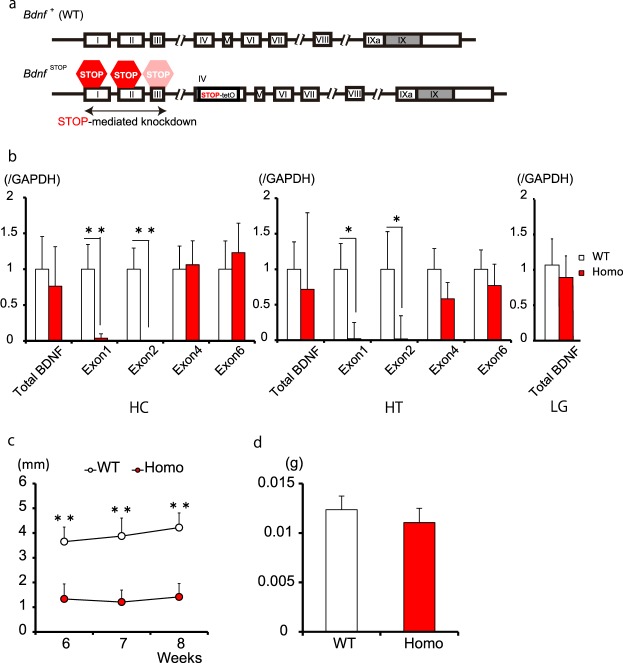
Bdnf isoforms 1 and 2 modulate basal tear secretion. (**a**) Model of BdnfSTOP/STOP mice (Homo). (**b**) Bdnf expression in the hippocampus (HC), hypothalamus (HT), and lacrimal gland (LG). BdnfSTOP/STOP mice showed no expression of exons 1 and 2 in the brain (n = 4–5; **P < 0.01, *P < 0.05, Student’s t-test). (**c**) Basal tear secretion of BdnfSTOP/STOP mice significantly decreased compared to control mice (n = 6–8; **P < 0.01, Student’s t-test). (**d**) LG weight was not changed in BdnfSTOP/STOP mice (n = 6–7).

**Figure 5:**
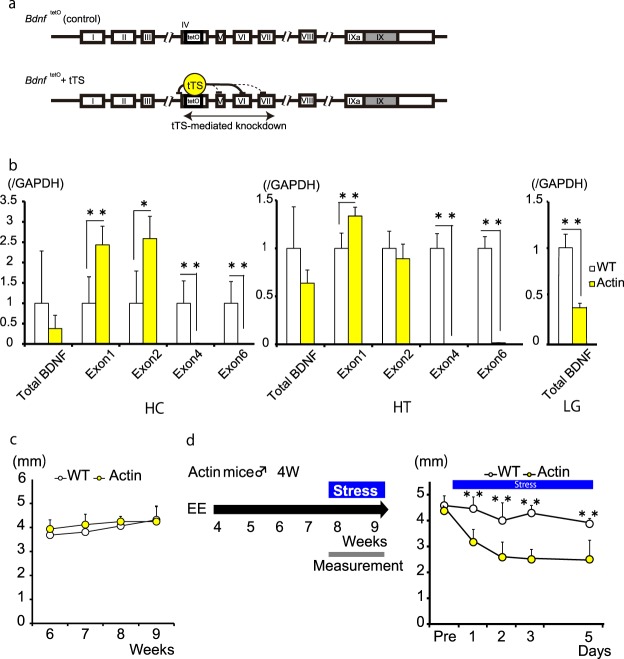
Bdnf isoforms 4 and 6 are necessary for enriched environment-related alleviation of stress-induced decreased tear secretion. (**a**) Model of Actin-tTS::BdnftetO/tetO mice (Actin). (**b**) Bdnf expression in the hippocampus (HC), hypothalamus (HT), and lacrimal gland (LG). Actin-tTS::BdnftetO/tetO mice showed no expression of exons 4 and 6 in the brain. Total Bdnf expression decreased significantly in the LG (n = 4–6; **P < 0.01, *P < 0.05, Student’s t-test). (**c**) Basal tear secretion in Actin-tTS::BdnftetO/tetO mice was similar to that in the control mice (n = 5–8). (**d**) Actin-tTS::BdnftetO/tetO mice did not maintain tear secretion under stress loading regardless of the presence of an enriched environment (EE) (n = 6; **P < 0.01, Student’s t-test).

We obtained *Bdnf* loss-of-function mutant mice in order to more comprehensively determine the causal relationship between BDNF levels and body condition. Using a unique gene knockdown strategy^32^, we were able to obtain two distinct *Bdnf* mutant lines and thus examine the roles of 2 *Bdnf* isoform clusters on tear secretion. One of these mutant lines is an isoform 1–3 cluster-targeted knockdown line (*Bdnf *^STOP/STOP^ homozygote), in which a STOP sequence blocks transcription of exons I–III (Fig. 4a). The other is an isoform 4–7 cluster-targeted line (*Actin*-tTS::*Bdnf *^tetO/tetO^) in which a tetracycline transcriptional silencer (tTS), present over a 4 kb radius of tetO sites in exon IV, mediates transcription silencing of exons IV–VII (Fig. 5a).

As expected, *Bdnf *^STOP/STOP^ mice showed no expression of *Bdnf* isoforms 1 and 2 in the brain and showed decreased total *Bdnf* mRNA levels (76% in the HC and 72% in the HT versus wildtype, Fig. 4b). Further, there was no difference in total *Bdnf* expression in the LG between the genotypes (Fig. 4b). This result was expected because isoform 6 should theoretically not be affected in *Bdnf *^STOP/STOP^ mice. We observed that basal tear secretion levels were significantly lower in *Bdnf *^STOP/STOP^ mice compared to those in WT mice (p < 0.01; Fig. 4c). LG weight in *Bdnf *^STOP/STOP^ mice was not significantly altered (Fig. 4d). These data indicate that loss of *Bdnf* isoforms 1 and 2 leads to decreased basal tear secretion.

We attempted to address the role of the various *Bdnf* isoforms on the relationship between EE and tear secretion. However, we found that *Bdnf *^STOP^ male homozygotes, and even heterozygotes, were very aggressive and attacked each other in group housing. This aggressive phenotype of *Bdnf *^STOP^ is consistent with that of exon 1 and exon 2 knock-out mice^28^. Therefore, we stopped the EE housing experiments in these mice for ethical reasons.

### Stress tolerance is partially mediated by *Bdnf* isoforms 4 and 6

The *Actin*-tTS::*Bdnf *^tetO/tetO^ mutant mice showed no expression of *Bdnf* isoforms 4 and 6 due to tTS-mediated gene silencing (Fig. 5a,b). These mice did show an increase in expression of *Bdnf* isoforms 1 and 2, probably due to a compensation effect. However, total *Bdnf* mRNA levels were still decreased (38% in the HC and 64% in the HT versus wildtype; Fig. 5b). Surprisingly, the amount of total *Bdnf* mRNA in the LG was unaffected even though tTS targeted isoform 6, the only isoform present in the LG (Fig. 5b). In the *Actin*-tTS::*Bdnf *^tetO/tetO^ mice, basal tear secretion was comparable to control mice, suggesting either that the isoform cluster 4–7 does not govern tear secretion or else that supplementary increases in isoforms 1 and 2 were able to normalize basal tear secretion levels.

Isoforms 4 and 6 knockdown mice (*Actin*-tTS::*Bdnf *^tetO/tetO^) were raised in group housing apart from isoforms 1 and 2 knockdown mice (*Bdnf *^STOP/STOP^ mice), enabling us to address the positive effect of EE on stress-induced DED-like symptoms. We housed *Actin*-tTS::*Bdnf *^tetO/tetO^ and control mice in EE cages at 4 weeks of age for 4 weeks and then exposed the mice to stress for 5 days. We observed no difference in basal tear secretion between wild type mice and *Actin*-tTS::*Bdnf *^tetO/tetO^ mice for the 4 weeks without stress loading (Fig. 5c). However, basal tear secretion in *Actin*-tTS::*Bdnf *^tetO/tetO^ mice significantly decreased in response to stress compared to that of control mice regardless of the presence of an EE (p < 0.01; Fig. 5d), indicating that isoforms 4 and 6 are necessary for the resilience and alleviation of DED-like symptoms observed in response to an EE.

Because it has been reported that BDNF expression is associated with mental disorders^23–25^, we analyzed *Bdnf *^STOP/STOP^ mice for behavioral problems. These mice did not show different behavior compared to wild type mice in behavior analysis, forced swim test (FST), elevated plus maze (EPM), or open field test (OFT; Supplementary Fig. 4). Similarly, the *Actin*-tTS::*Bdnf *^tetO/tetO^ mice did not show different behavior compared to the wild type mice in behavior analysis, FST, EPM, or OFT (Supplementary Fig. 5). These results suggest that the effects of an EE on stress-loading require expression of exons IV and VI in the brain and that *Bdnf* isoforms 4 and 6 may contribute to stress tolerance for tear secretion in mice.

## Discussion

In the current study, we found that basal tear secretion decreases in response to stress in mice. Additionally, we observed that housing mice in an EE suppresses this decrease and allows for a quicker recovery from a stressed state. Because brain *Bdnf* gene expression in mice decreases in response to stress and increases in response to an EE, we conducted a *Bdnf* loss-of-function study to investigate the mechanism by which BDNF regulates basal tear secretion. Our results show that *Bdnf* knockdown mice have decreased basal tear secretion. No improvement was observed in these knockdown mice when recovering from a stressed state in an EE. These results indicate that *Bdnf* plays an important role in basal tear secretion in mice.

Many mouse models have been employed to investigate the symptoms of DED, and several interventions have been explored. For example, a transgenic line was generated from db/db mice (db/db-CDK4R24C) that expresses a constitutively active form of cyclin-dependent kinase 4 (CDK4/R24C) under the control of an insulin promoter to model diabetic dry eyes^33^. Additionally, experimental dry eye has been induced in interleukin-1 receptor-1 (IL-1R1)-deficient (knock-out) mice^34^ and in mice with surgically-excised lacrimal glands^35^. Moreover, one study used a controlled environment chamber with low humidity to induce DED^36^. However, each of these models has specific limitations. Transgenic mouse models show whole-body changes, such as decreased body weight, and mouse models with a surgically excised LG show an increased inflammatory response. Further, the controlled environment chamber only models dry eye caused by low humidity, rather than dry eye caused by stress or VDT use. Our methods, blowing air over the eyes and restraining the body, did not cause whole-body adverse events. More importantly, our mouse model closely resembles the development of dry eye symptoms due to the use of VDTs^5^. Use of a VDT has been shown to lead to decreased blinking and a more fragile tear film on the cornea^37^. Blowing air over the eyes in mice would be expected to weaken the tear film. In addition, people using a VDT tend to be more physically sedentary and restrained to a desk. We thus propose that our mouse model, which uses blowing and restraint, specifically mimics the onset of dry eye due to use of a VDT.

Currently, dry eye symptoms are treated with supplemental eye drops that do not target the source of the condition. Therefore, there is a demand for an intervention that targets the cause of decreased basal tear secretion. Two major factors that decrease treatment effectiveness are a lack of exercise (being sedentary), and a low level of well-being^38^. Intervention to improve these factors may thus alleviate the symptoms of dry eye in humans, and we employed an animal model to examine the effect of an EE, which is an environment with added sensory, cognitive, and social stimuli compared to a standard environment (SE)^39^. These factors can increase exercise and can model increased levels of well-being. Simply increasing voluntarily exercise did not result in any changes in tear secretion in our mouse model, but holding dry eye model mice in an EE led to an improvement in tear secretion. These results imply that exercise habits and increased well-being are likely to improve symptoms rather than just exercise alone^38,40^.

Mice held in an EE experience varied anatomical, physiological, and genetic changes. For example, several publications have reported an increase in *Bdnf* gene expression after holding mice in an EE^41–43^. Our experiments with *Bdnf* loss-of-function mice provide evidence that lowered *Bdnf* expression inhibits EE-associated recovery, suggesting that *Bdnf* gene induction after EE housing plays a key role in recovery from decreased tear secretion. Indeed, clinical research has shown that gene polymorphisms associated with decreased BDNF release is related to dry eye syndrome^26^, supporting the idea that BDNF expression has a positive effect on basal tear secretions in human. Because neuropsychiatric conditions such as depression are known to be associated with decreased BDNF expression^44–46^, the comorbidity between dry eye syndrome and neuropsychiatric disorder may account for the observed BDNF dysregulation. Further research is necessary, but it is possible that DED reflects underlying neuropsychiatric conditions.

Here, we report two distinct loss-of-function manipulations of *Bdnf* mRNA isoform clusters. The first method uses a STOP cassette knock-in to target isoform cluster 1–3. Although single isoform knock-out mice have been reported previously^47^, double and triple isoform knock-out mice cannot be generated by breeding single isoform knock-out mice. Hence, isoform cluster knock-out by a STOP cassette allows for the discovery of new phenotypes. The other method employed in this study used a tTS-tetO system. Specifically, a TetO sequence was knocked into exon IV, and tetO-tethering tTS blocked transcription over a 3 kilobase radius, which contains exons V–VII. This resulted in an isoform 4–7 cluster knockdown. In the future, we hope to investigate a timing-specific and reversible isoform 4–7 knockdown using doxycycline administration^48^ as well as an organ/tissue/cell-specific reversible knockdown using a cell type-specific tTS line, such as *CamK2*-tTS^49,50^.

The present study demonstrates that an enriched environment effectively prevents and improves dry eye in mice. Additionally, our results indicate that BDNF is a key factor in dry eye pathogenesis, which we hope will be useful for future research on the relationship between BDNF and dry eye.

## Methods

### Ethical statement

All animal procedures were conducted in accordance with the National Institutes of Health Guide for the Care and Use of Laboratory Animals and approved by the Keio University Institutional Animal Care and Use Committee. Male C57BL/6JJcl mice were obtained from CLEA Japan, Inc. (Tokyo, Japan). *Bdnf* mutant mice were generated as described below. All mice received food and water ad libitum and were housed in an atmosphere-controlled room with 12-hour light/dark cycles.

### Stress loading

Mice were restrained by placing them into a 50 mL plastic tube (IWAKI, Japan) as a psychological stressor. The cap of the tube had 7 holes, each with a 2-mm diameter, and mice faced the cap. Mice were exposed to constant low humidity air flow aimed at the face as a mechanical stressor. The air flow was produced by an electric fan (Funai, Japan). Stress loading occurred for 4 hours unless noted otherwise.

### Environmental enrichment housing

EE housing started at 4 weeks of age and consisted of a larger cage (39 cm × 53 cm × 18 cm), one running wheel to increase physical exercise, an assortment of toys (an igloo, a dome, tunnels, and wood objects) to increase perception and mental exercise, and 6 company mice with nesting material to increase social interaction. The toys were changed twice per week. SE housing consisted of regular-sized cages (22 cm × 33 cm × 14 cm) housing single mice.

### Measurement of tear secretion volume (cotton thread test)

The mean weight-adjusted aqueous tear secretion quantity was measured using phenol red-impregnated cotton threads (ZoneQuick, Showa Yakuhin Kako, Tokyo, Japan) without anesthesia. The cotton threads were placed at the lateral canthus for 15 seconds, and the wetting length was measured using a ruler purchased from Showa Yakuhin Kako for measuring 1-mm units and a scale purchased from Shinwa Sokutei (Tsumabe, Japan) for measuring lengths less than 1 mm. Measurements were made to the nearest 0.1 mm.

### Ocular surface vital staining assessment

Alterations of corneal vital staining were assessed 2 minutes after fluorescein dye instillation. Corneal vital staining results were recorded by a digital camera-equipped microscope using the same settings (exposure time) for all mice.

### Open field test

An OFT was performed in a 36 cm × 36 cm × 26 cm white-colored box for 30 min. The brightness of the field was 50 lux. Each mouse was recorded by a web camera for 5 minutes, and the recorded data were analyzed automatically using a software package (Any-Maze, Stoelting, Wood Dale, IL).

### Elevated plus maze

The EPM test apparatus consisted of 2 open arms and 2 closed arms (25 cm × 5 cm) that extended from a central platform (5 cm × 5 cm). The closed arms were surrounded by walls that were 40 cm in height. The maze was elevated 40 cm above the floor, and the room lights (4100 lux) were turned on during testing. Mice typically avoid the open arms because they innately dislike open space, and anxiolytic agents increase the time spent in open arms. That is, a decrease in the time spent in the open arms indicates an increase in anxiety. The distance travelled in the maze is used to quantify locomotor activity. The test was initiated by placing the mouse on the central platform facing an open arm, and recording began once the mouse entered a closed arm. If a mouse failed to enter a closed arm after 1 min, data from that mouse were excluded from the analysis (one mouse was excluded). Each mouse was recorded by a web camera over a 5 min period, and the recorded data were analyzed automatically using a software package (Any-Maze, Stoelting).

### Forced swim test

A 6-minute FST was applied, and behaviors were video-recorded and scored later by a researcher who was blind to experimental conditions. Only the last 4 minutes of behavior were analyzed, and each minute was categorized as either immobile (i.e., the mouse was completely still in the water except for isolated movements to right itself), or climbing (i.e., the mouse was moving all four legs with its body aligned vertically in the water).

### RNA extraction and qPCR

Mice were sacrificed by cervical dislocation, and brain and LG tissues were collected. Total RNA was isolatsed from the HT, HC, prefrontal cortex, and LG using TRIzol (Life Technologies, Carlsbad, CA). Genomic DNA was removed from total RNA using gDNA remover (TOYOBO, Osaka, Japan). cDNA was synthesized from the resulting genomic DNA using ReverTra Ace qPCR RT Master Mix (TOYOBO, Osaka, Japan). Quantitative PCR was performed with a TaqMan probe (Life Technologies) and Step One Plus (Thermo Fisher Scientific, Waltham, MA). Primer and TaqMan sequences are shown in Supplementary Table S1 online. PCR efficiencies of the *Bdnf* primers were examined by a standard curve of serial-diluted cDNA. Individual mRNA levels were normalized using *Gapdh* mRNA content.

### Generation of *Bdnf* mutant mice

We constructed a *Bndf* gene targeting vector in which the following elements were connected in tandem: a 10 kb 5-prime homology arm, a 3.5 kb Neo-STOP-tetO cassette^31^, a 1.9 kb 3-prime homology arm, and a diphtheria toxin A subunit (Supplementary Fig. 6). The Neo-STOP-tetO cassette was comprised of a 1.7 kb PGK-Neo, a 1.3 kb STOP sequence, and a 0.5 kb tetO site. The targeting vector was designed to insert the Neo-STOP-tetO cassette into exon IV. *Bdnf* exon IV consists of 339 bases, and the first ATG appears at 74 nt, while the last ATG appears at 310 nt. The Neo-STOP-tetO cassette was used to replace nucleotides 74–310 within exon IV. We used 129 SvEv-derived embryonic cells for the homologous recombination. We obtained 4 recombined clones out of 80 G418-resistant clones. Germline transmitted offspring were established as *Bdnf* ^STOP-tetO^ knock-in mice (Supplementary Fig. 6). *Bdnf *^STOP-tetO^ mice were crossed with ROSA-Flpe mice, FRT flanking Neo-STOP sequences were removed (Supplementary Fig. 6), and Bdnf ^tetO^ knock-in mice were generated. Bdnf ^tetO^ knock-in mice were cross-bred with *Actin*-tTS^51^ mice to subsequently generate Actin-tTS hemizygous and Bdnf tetO knock-in homozygous mice (*Actin*-tTS::*Bdnf *^tetO/tetO^). We considered Bdnf STOP-tetO homozygotes (*Bdnf *^STOP/STOP^) and *Actin*-tTS::*Bdnf *^tetO/tetO^ as *Bdnf* loss-of-function mice.

The following sets of primers were used for genotyping: ttsP1 (5′-TTG ATC ACC AAG GTG CAG AG-3′) and ttsP2 (5′-CAG GGC TCT CCC TTC TC-3′) were used for the *Actin*-tTS allele and yielded a band of approximately 400 bp; BDNFup (5′-CAG CGT GGA GCC CTC TCG TG-3′) and PGKproL1 (5′-GTT GGC GCC TAC CGG TGG ATG TGG AAT GTG-3′) yielded an approximately 340-bp band from the *Bdnf *^STOP-tetO^ knock-in allele; and tetOup (5′-AGC AGA GCT CGT TTA GTG AAC CGT-3′) and BDNFlow (5′-TTG CGC CCT GAC CTC TCC GG-3′) yielded an approximately 380-bp band from the *Bdnf *^STOP-tetO^ knock-in and *Bdnf *^tetO^ knock-in alleles. BDNFup and BDNFlow yielded a 570-bp band from the wild type allele and a 900-bp band from the *Bdnf *^tetO^ knock-in allele (Supplementary Fig. 6).

### Statistical analysis

Sample sizes were determined on the basis of pilot experiments and previous experience from similar experiments. We used an F-test to determine whether the data had the same variances. As all the data were determined to be normally distributed, parametric statistics were used throughout. All data were analyzed by Student’s t-test, one-way ANOVA, or one-way ANOVA with post-hoc Tukey’s test using SPSS software version 25 (IBM, Armonk, New York, USA). Statistical significance was established at a threshold of P < 0.05. The statistical test used for each experiment is stated in the corresponding figure legend.

## Supplementary information

all supplementary information

## Data Availability

The datasets generated during the current study are available from the corresponding author on reasonable request.

## References

[1] Tsubota K, Nakamori K (1995). Effects of ocular surface area and blink rate on tear dynamics. Arch. Ophthalmol..

[2] Nakamori K, Odawara M, Nakajima T, Mizutani T, Tsubota K (1997). Blinking is controlled primarily by ocular surface conditions. Am. J. Ophthalmol..

[3] Uchino M (2008). Prevalence of dry eye disease among Japanese visual display terminal users. Ophthalmology..

[4] Kojima T (2011). The impact of contact lens wear and visual display terminal work on ocular surface and tear functions in office workers. Am. J. Ophthalmol..

[5] Uchino M (2013). Prevalence of dry eye disease and its risk factors in visual display terminal users: the Osaka study. Am. J. Ophthalmol..

[6] Management and therapy of dry eye disease (2007). report of the Management and Therapy Subcommittee of the International Dry Eye Workshop. Ocul. Surf..

[7] Miljanovic B, Dana R, Sullivan DA, Schaumberg DA (2007). Impact of dry eye syndrome on vision-related quality of life. Am. J. Ophthalmol..

[8] Dogru M, Katakami C, Inoue M (2001). Tear function and ocular surface changes in noninsulin-dependent diabetes mellitus. Ophthalmology..

[9] Hallak JA, Tibrewal S, Jain S (2015). Depressive symptoms in dry eye disease patients: A case-control study using the beck depression inventory. Cornea..

[10] Tiskaoglu NS (2017). Dry eye disease in patients with newly diagnosed depressive disorder. Curr. Eye. Res..

[11] Szakáts I, Sebestyén M, Németh J, Birkás E, Purebl G (2016). The role of health anxiety and depressive symptoms in dry eye disease. Curr. Eye Res..

[12] LI M, Gong L, Sun X, Chapin WJ (2011). Anxiety and depression in patients with dry eye syndrome. Curr. Eye. Res..

[13] Wan KH, Chen LJ, Young AL (2016). Depression and anxiety in dry eye disease: a systematic review and meta-analysis. Eye..

[14] Ayaki M (2016). Sleep and mood disorders in women with dry eye disease. Sci. Rep..

[15] Ayaki M, Kawashima M, Negishi K, Tsubota K (2015). High prevalence of sleep and mood disorders in dry eye patients: survey of 1,000 eye clinic visitors. Neuropsychiatr. Dis. Treat..

[16] Kawashima M (2016). The association of sleep quality with dry eye disease: the Osaka study. Clin. Ophthalmol..

[17] Nakamura S (2010). Lacrimal hypofunction as a new mechanism of dry eye in visual display terminal users. PloS One..

[18] Jess N, Anthony JH (2006). Enriched environments, experience-dependent plasticity and disorders of the nervous system. Nature..

[19] Guohua L (2015). Enriched environment inhibits mouse pancreatic cancer growth and down-regulates the expression of mitochondria related genes in cancer cells. Sci. Rep..

[20] Song Y (2017). Enriching the housing environment for mice enhances their NK cell antitumor immunity via sympathetic nerve dependent regulation of NKG2D and CCR5. Cancer Res..

[21] Ickes BR (2000). Long-term environmental enrichment leads to regional increases in neurotrophin levels in rat brain. Exp. Neurol..

[22] Young D, Lawlor PA, Leone P, Dragunow M, During MJ (1999). Environmental enrichment inhibits spontaneous apoptosis, prevents seizures and is neuroprotective. Nat. Med..

[23] Keri M, Manji H, Lu B (2007). New insights into BDNF function in depression and anxiety. Nat. Neurosci..

[24] Hariri AR (2003). Brain-derived neurotrophic factor val66met polymorphism affects human memory related hippocampal activity and predicts memory performance. J. Neurosci..

[25] Notaras M, Hill R, van den Buuse M (2015). The BDNF gene Val66Met polymorphism as a modifier of psychiatric disorder susceptibility: progress and controversy. Mol. Psychiatry..

[26] Hallak JA, Tibrewal S, Mohindra N, Gao X, Jain S (2015). Single nucleotide polymorphisms in the BDNF, VDR, and DNASE 1 genes in dry eye disease patients: A case-control study. Invest. Ophthalmol. Vis. Sci..

[27] Aid T, Kazantseva A, Piirsoo M, Palm K, Timmusk T (2007). Mouse and rat BDNF gene structure and expression revised. J. Neurosci. Res..

[28] Pruunsild P, Kazantseva A, Aid T, Palm K, Timmusk T (2007). Dissecting the human BDNF locus: bidirectional transcription, complex splicing, and multiple promoters. Genomics..

[29] Maynard KR (2016). Functional role of BDNF production from unique promoters in aggression and serotonin signaling. Neuropsychopharmacology..

[30] Sakata K (2009). Critical role of promoter IV-driven BDNF transcription in GABAergic transmission and synaptic plasticity in the prefrontal cortex. Proc. Natl. Acad. Sci. USA.

[31] Simsek C, Kojima T, Dogru M, Tsubota K (2018). Alterations of murine subbasal corneal nerves after environmental dry eye stress. Invest. Ophthalmol. Vis. Sci..

[32] Tanaka KF (2010). Flexible accelerated STOP Tetracycline Operator-knockin (FAST): a versatile and efficient new gene modulating system. Biol. Psychiatry..

[33] Zagon IS (2014). Ocular surface abnormalities related to type 2 diabetes are reversed by the opioid antagonist naltrexone. Clin Exp Ophthalmol..

[34] Narayanan S, Sassani JW, Immonen JA, McLaughlin PJ (2008). Interleukin-1 receptor-1-deficient mice show attenuated production of ocular surface inflammatory cytokines in experimental dry eye. Cornea..

[35] Fuchikami M, Morinobu S, Kurata A, Yamamoto S, Yamawaki S (2009). Single immobilization stress differentially alters the expression profile of transcripts of the brain-derived neurotrophic factor (BDNF) gene and histone acetylation at its promoters in the rat hippocampus. Int. J. Neuropsychopharmacol..

[36] Barabino S (2005). The controlled-environment chamber: a new mouse model of dry eye. Invest. Ophthalmol. Vis. Sci..

[37] Hirota M, Kawamorita T, Shibata Y, Yamamoto S (2013). Effect of incomplete blinking on tear film stability. Optom. Vis. Sci..

[38] Kawashima M (2015). Association between subjective happiness and dry eye disease: a new perspective from the Osaka study. PloS One..

[39] van Praag H, Kempermann G, Gage FH (2000). Neural consequences of environmental enrichment. Nat. Rev. Neurosci..

[40] Sano K, Kawashima M, Takechi S, Mimura M, Tsubota K (2018). Exercise program improved subjective dry eye symptoms for office workers. Clin. Ophthalmol..

[41] Falkenberg T (1992). Increased expression of brain-derived neurotrophic factor mRNA in rat is associated with improved spatial memory and enriched environment. Neurosci. Lett..

[42] Cao W (2014). Early enriched environment induces an increased conversion of proBDNF to BDNF in the adult rat’s hippocampus. Behav. Brain. Res..

[43] Meng FT (2015). Beneficial effects of enriched environment on behaviors were correlated with decreased estrogen and increased BDNF in the hippocampus of male mice. Neuro. Endocrinol. Lett..

[44] Angelucci F, Brenè S, Mathé AA (2005). BDNF in schizophrenia, depression and corresponding animal models. Mol. Psychiatry..

[45] Shirayama Y, Chen AC, Nakagawa S, Russell DS, Duman RS (2002). Brain-derived neurotrophic factor produces antidepressant effects in behavioral models of depression. J. Neurosci..

[46] Karege F (2002). Decreased serum brain-derived neurotrophic factor levels in major depressed patients. Psychiatry Res..

[47] Nelson RJ, Young K (1998). Behavior in Mice with Targeted Disruption of Single Genes. Neurosci. Biobehav. Rev..

[48] Watabe T (2017). Time-controllable Nkcc1 knockdown replicates reversible hearing loss in postnatal mice. Sci. Rep..

[49] Richardson-Jones JW (2011). Serotonin-1A autoreceptors are necessary and sufficient for the normal formation of circuits underlying innate anxiety. J. Neurosci..

[50] Nautiyal KM (2015). Distinct Circuits Underlie the Effects of 5-HT1B Receptors on Aggression and Impulsivity. Neuron..

[51] Mallo M, Kanzler B, Ohnemus S (2003). Reversible gene inactivation in the mouse. Genomics..

