# Supplementary material for: Enriched environment alleviates stress-induced dry-eye through the BDNF axis
Source: Sci Rep. 2019 Mar 4;9:3422. doi: 10.1038/s41598-019-39467-w (PMC6399317; doi:10.1038/s41598-019-39467-w)
Supplement: Supplementary file 1 — all supplementary information [file 41598_2019_39467_MOESM1_ESM.pdf]

Enriched environment alleviates stress-induced dry-eye through the BDNF axis

Kokoro Sano[1], Motoko Kawashima[1], Toshihiro Imada[1], Toru Suzuki[2], Shigeru Nakamura[1], Masaru Mimura[2], Kenji F Tanaka[2]*, Kazuo Tsubota[1]*

1. Department of Ophthalmology, Keio University School of Medicine, Tokyo 160-8582, Japan.
2. Department of Neuropsychiatry, Keio University School of Medicine, Tokyo 160-8582, Japan.

Correspondence to:

Kenji F. Tanaka and Kazuo Tsubota

Keio University School of Medicine,

35 Shinanomachi, Shinjuku, Tokyo, 160-8582, Japan

kftanaka@keio.jp Tel: +81 3 5363 3934

tsubota@z3.keio.jp Tel; +81 3 5363 3219

Supplementary figure legends

Supplementary Figure 1 | Stress loading does not change lacrimal gland weight or body weight.

Mice did not show changes in **(a)** lacrimal gland weight or **(b)** body weight (n=6).

Supplementary Figure 2 | The effects of an enriched environment on body weight and stress-induced decreased tear secretion.

Mice did not show any changes in **(a)** lacrimal gland weight or **(b)** body weight (n=5–6). **(c)** The presence of stress loading did not affect fluorescein vital staining in mice housed in an enriched environment (EE; n=3). **(d)** Tear secretion decreased when EE housing was discontinued during stress loading (n=6; **P<0.01,*P<0.05, Student's t-test). **e** EE housing led to tear secretion recovery, even during stress loading (n=6; **P<0.01,*P<0.05, Student's t-test).

Supplementary Figure 3 |*Bdnf* expression in the lacrimal gland (LG).

There were no significant differences in *Bdnf* expression in the LG observed **(a)** after stress loading, **(b)** with an EE, or **(c)** with stress loading and an EE (n=5–6).

Supplementary Figure 4 | Behavioral analysis of *Bdnf*$^{STOP/STOP}$ mice.

(a) Forced swim test (FST; n=5–7) **(b)** Elevated plus maze (EPM; n=5–7) **(c)** Open field test (OFT; n=5–7).

Supplementary Figure 5 | Behavior analysis of *Actin*-tTS::*Bdnf*$^{tetO/tetO}$ mice. **(a)** Forced swim test (FST; n=6) **(b)** Elevated plus maze (EPM; n=6) **(c)** Open field test (OFT;

n=6)

Supplementary Figure 6 | Generation of *Bdnf* mutant mice.

Supplementary table legend

Supplementary Table S1 | Sequences of PCR primers and TaqMan probes.

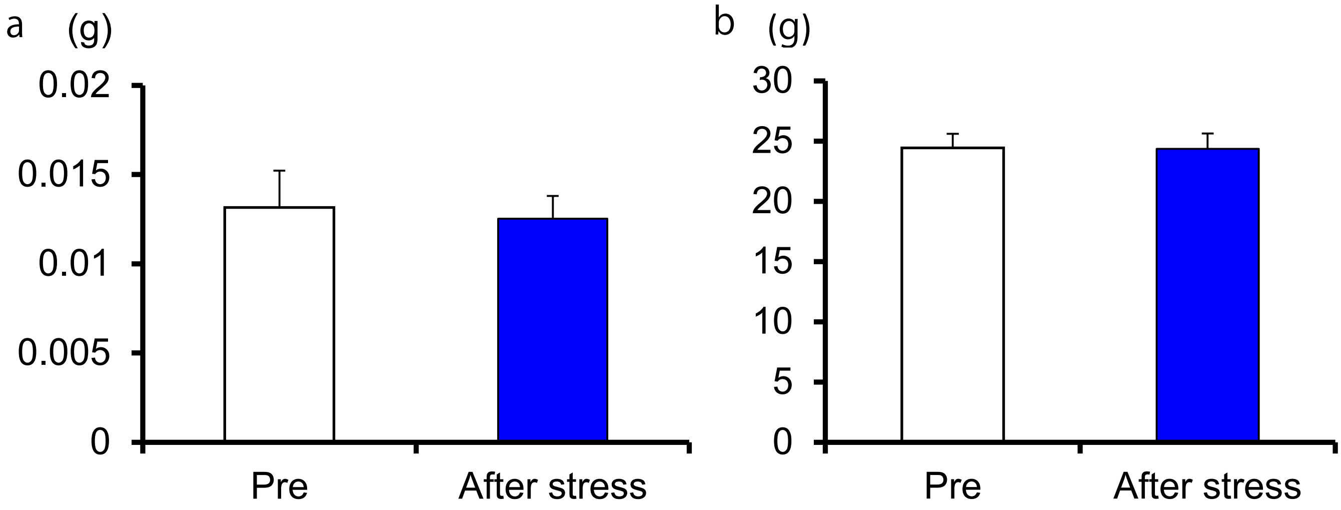

Supplementary Figure-1

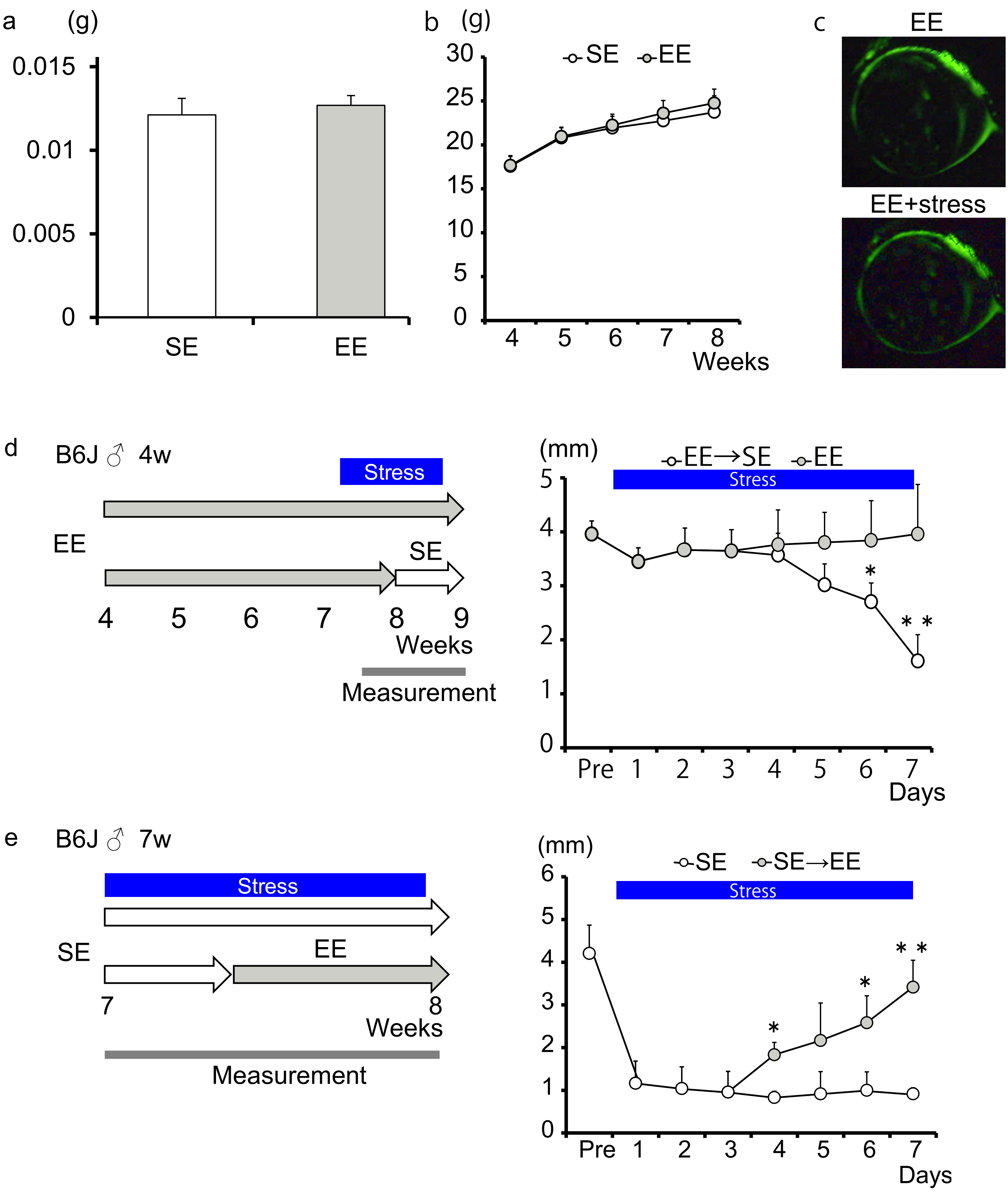

Supplementary Figure-2

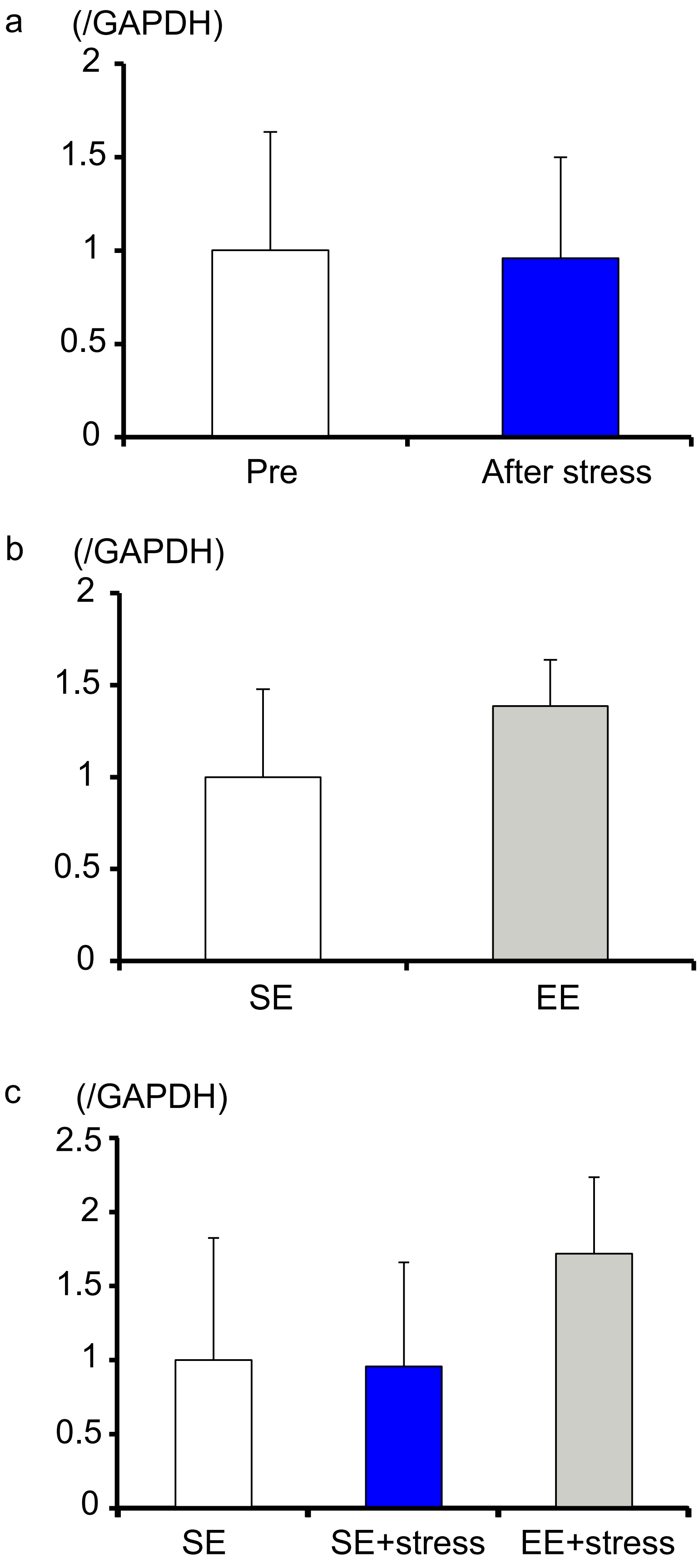

Supplementary Figure-3

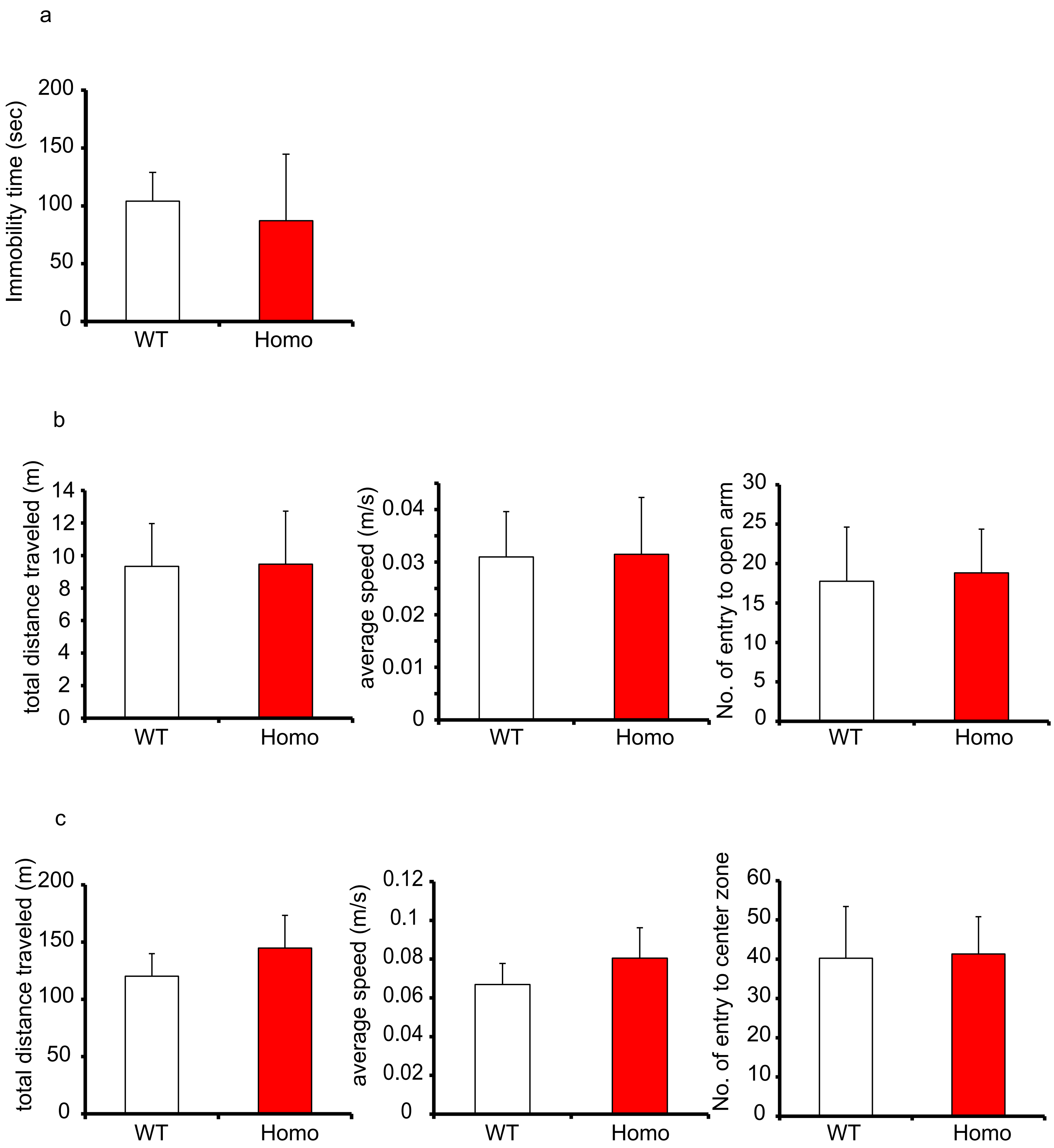

Supplementary Figure-4

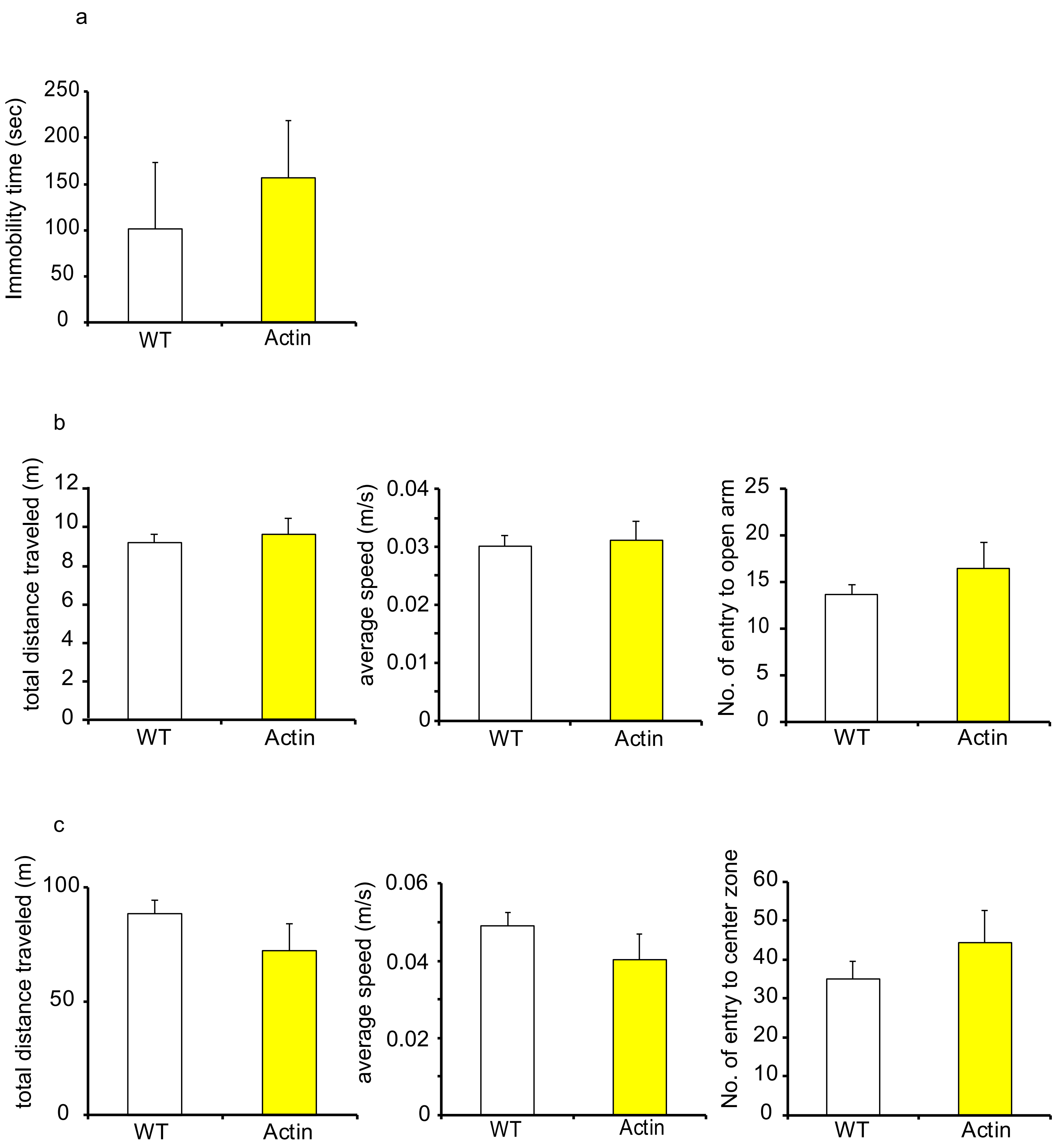

Supplementary Figure-5

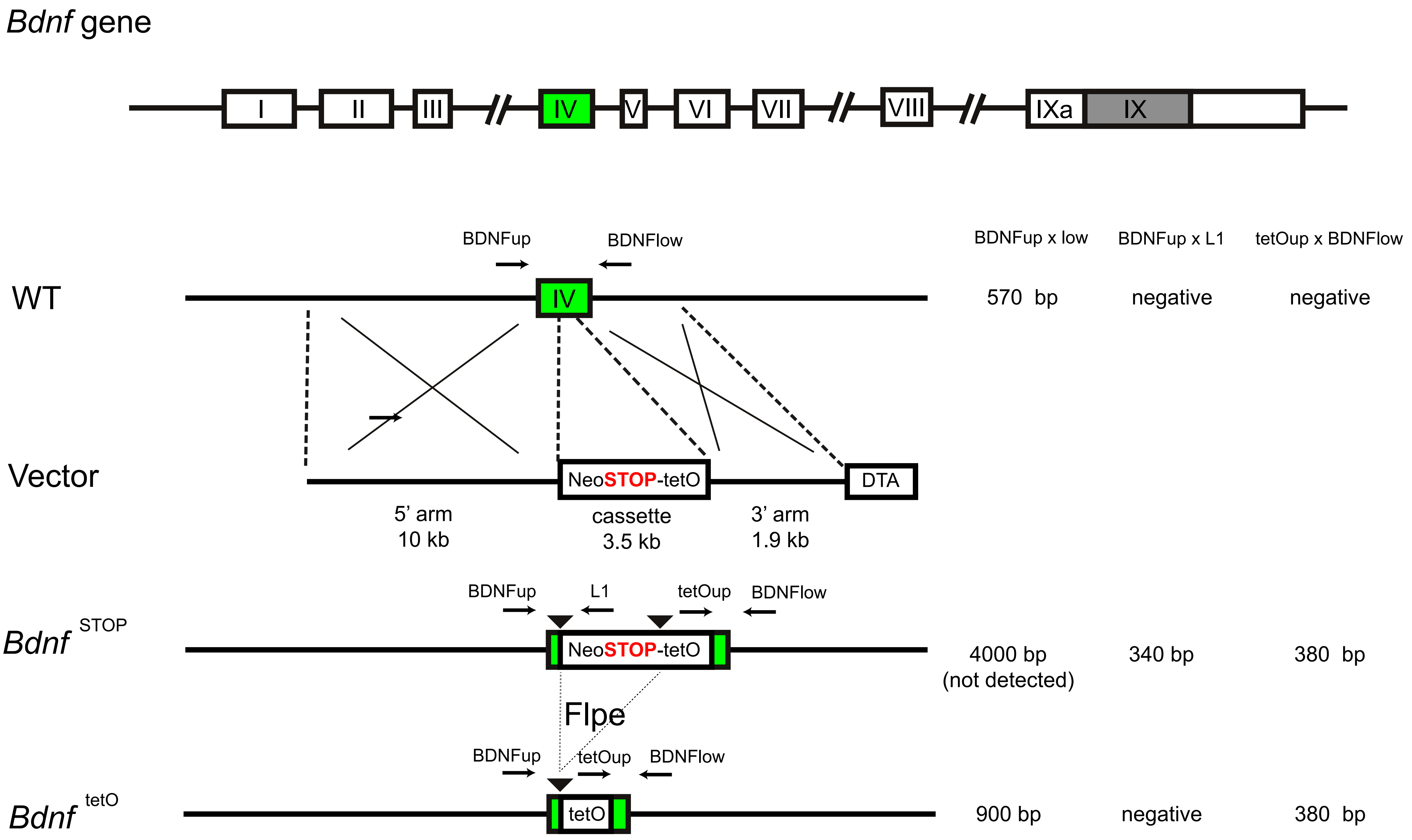

Supplementary Figure-6

Gene/Allele	Forward Primer (5' to 3')	Reverse Primer (5' to 3')	Probe Sequence (5' to 3')*
BDNF Exon 1	CACATTACCTTCCTGCATCTGTTG	ACCATAGTAAGGAAAAGGATGGTCAT	6FAM-AAGCCACAATGTTCCACCA
BDNF Exon 2	TTGGGAAATGCAAGTGTTTATCA	CGAAGTATGAAATAACCATAGTAAGGAAAA	6FAM-CCGCAAAGAAGTTCCACCAG
BDNF Exon 4	CTGCCTTGATGTTTACTTTGACAAG	ACCATAGTAAGGAAAAGGATGGTCAT	6FAM-TGACTGAAAAAGTTCCACCAGG
BDNF Exon 6	CAGAAGCGTGACAACAATGTGA	ACCATAGTAAGGAAAAGGATGGTCAT	6FAM-ACCCTGAGTTCCACCAGG
			**Taqman Probe ID (Life Technologies)**
Gapdh			Mm99999915_g1
Total BDNF			Mm04230607_s1
Htr2a			Mm00555764_m1
Pvalb			Mm00443100_m1

Supplementary Table-1
